# Reflectance confocal microscopy of clear cell acanthoma: A novel insight to avoid invasive procedures

**DOI:** 10.1111/srt.13901

**Published:** 2024-08-02

**Authors:** Federico Venturi, Emi Dika

**Affiliations:** ^1^ Oncologic Dermatology Unit IRCCS Azienda Ospedaliero‐Universitaria di Bologna Bologna Italy; ^2^ Department of Medical and Surgical Sciences (DIMEC) Alma Mater Studiorum University of Bologna Bologna Italy

Clear cell acanthoma (CCA) is a benign squamous lesion composed of keratinocytes with pale to clear cytoplasm.^1^ Typically located on the lower extremities, CCA presents as solitary, reddish‐brown to skin‐colored papule or nodule. Dermoscopic examination of CCA can provide valuable insights into its characteristic features, aiding in the differential diagnosis from other skin conditions such as seborrheic keratosis, Bowen disease, porocarcinoma, and atypical spitz tumors.^2^, ^3^, ^4^ In most cases, dermoscopic examination reveals dotted vessels arranged linearly in a pattern that resembles a string of pearls.^5^ However, histopathological confirmation remains the gold standard for diagnosis. Reflectance confocal microscopy (RCM) is a valuable tool in dermatology for non‐invasive, in vivo imaging of skin lesions with cellular resolution. Its ability to provide real‐time visualization of the skin's microarchitecture makes it an invaluable adjunct to clinical examination and histopathological analysis.^6^ Regarding CCA, RCM may provide valuable insights in the diagnostic setting, avoiding unnecessary invasive procedures. To date, only two reports have fully addressed this finding.^7^, ^8^ Our aim with this letter is to present specific RCM features of CCA and a new parameter in order to improve diagnostic accuracy and avoid unnecessary invasive procedures. Recently, a 60‐year‐old patient was referred to our clinic for a pinkish nodular lesion of the right leg recently appeared. The dermoscopic evaluation revealed a pattern of vessels forming a necklacelike distribution (Figure 1A). RCM examination with Vivascope 3000 (MAVIG GmbH, Munich, Lucid‐Tech Inc., Henrietta, NY, USA) (4 × 4 mm mosaic) was carried out, showing a sharp lateral circumscription by collarette of hyperreflective hyperkeratotic cells, epidermal disarray together with dilated blood vessels expanding dermal papillae and extending into the spinous layer confirming the clinical diagnosis of CCA (Figure 1B). The examination performed with Vivascope 1500 (0.5 × 0.5 mm) at the level of the upper dermis displayed the presence of an inflammatory cell infiltrate, epidermal disarray, and parakeratosis and a detail of the dilated u‐shaped vessels (Figure 1C). As far as we know, solitary pink lesions represent a clinical challenge even for expert dermatologists as they typically display fewer features than pigmented ones and often require a biopsy to exclude malignancy. Therefore, RCM integration in our clinical practice is particularly useful in this specific setting as it allows the identification of features previously only seen in histopathology with no need for surgical excision or dermoscopic monitoring.^9^, ^10^ Even if categorized as a benign tumor, CCA is often subjected to excisional biopsy to rule out malignancies with possible complications and psychological impairment related to the surgical procedure. In combination with dermoscopy, RCM allows the detection of specific features and patterns and leads to the correct diagnosis. In the case of CCA, it strictly correlates with histopathology, by enhancing the identification of a sharp demarcation of the lateral edges by a collarette of hyperreflective hyperkeratotic cells, inflammatory cells infiltrate, and a specific vascular pattern defined by the presence of dilated u‐shaped vessels. Differential diagnosis with Bowen disease as well as other malignancies that can occur on the lower limbs such as atypical spitz tumors and porocarcinomas is so ruled out. In conclusion, despite the difficult clinical recognition, CCA displays dermoscopically the “string of pearls” vessel pattern which, combined with the stereotypical and specific RCM appearance defined by the hyperreflective collarette and the u‐shaped vessels allows a confident diagnosis with no need for further investigations.

**FIGURE 1:**
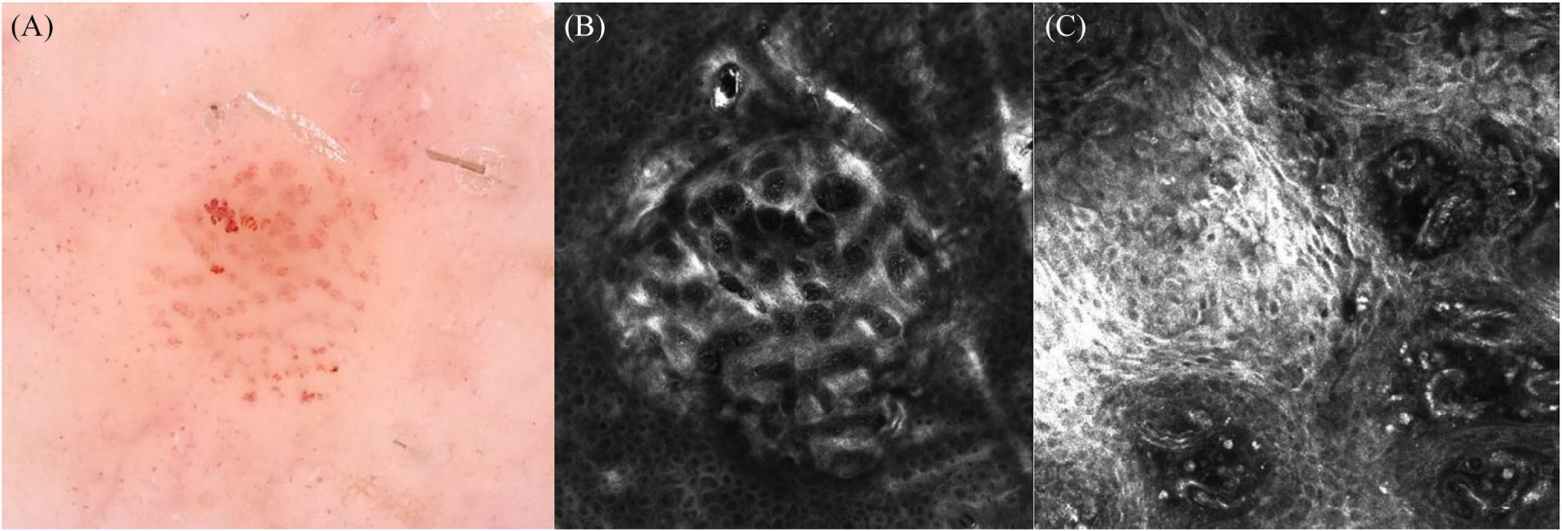
Dermoscopic and RCM features of clear cell acanthoma. Dermoscopic evaluation revealed a pattern of vessels forming a necklacelike distribution (A); RCM examination with Vivascope 3000 (4 × 4 mm mosaic) showed a sharp lateral circumscription by a collarette of hyperreflective hyperkeratotic cells, epidermal disarray together with dilated blood vessels expanding dermal papillae and extending into the spinous layer (B); RCM performed with Vivascope 1500 (0.5 × 0.5 mm) at the level of the upper dermis displayed the presence of an inflammatory cell infiltrate, epidermal disarray, and parakeratosis and a detail of the dilated u‐shaped vessels (C).

## CONFLICT OF INTEREST STATEMENT

The authors have no relevant financial or non‐financial interests to disclose.

## PATIENTS CONSENT

Patient was informed about the use of clinical information according to the Declaration of Helsinki principles and photos for a publication intent. The informed consent was appropriately obtained during the medical examination.
